# Risk factors for stroke after total aortic arch replacement using the frozen elephant trunk technique

**DOI:** 10.1093/icvts/ivac013

**Published:** 2022-01-29

**Authors:** Tim Berger, Maximilian Kreibich, Felix Mueller, Lara Breurer-Kellner, Bartosz Rylski, Stoyan Kondov, Holger Schröfel, Clarence Pingpoh, Friedhelm Beyersdorf, Matthias Siepe, Martin Czerny

**Affiliations:** 1 Department of Cardiovascular Surgery, Faculty of Medicine, University Heart Centre, University Hospital Freiburg, Albert-Ludwigs-University of Freiburg, Freiburg, Germany; 2 Department of Neurology, Faculty of Medicine, University Hospital Freiburg, Albert-Ludwigs-University of Freiburg, Freiburg, Germany

**Keywords:** Aortic dissection, Frozen elephant trunk, Redo arch surgery, Arch replacement

## Abstract

**OBJECTIVES:**

This study aimed to analyse risk factors for postoperative stroke, evaluate the underlying mechanisms and report on outcomes of patients suffering a postoperative stroke after total aortic arch replacement using the frozen elephant trunk technique.

**METHODS:**

Two-hundred and fifty patients underwent total aortic arch replacement via the frozen elephant trunk technique between March 2013 and November 2020 for acute and chronic aortic pathologies. Postoperative strokes were evaluated interdisciplinarily by a cardiac surgeon, neurologist and radiologist, and subclassified to each’s cerebral territory. We conducted a logistic regression analysis to identify any predictors for postoperative stroke.

**RESULTS:**

Overall in-hospital was mortality 10% (25 patients, 11 with a stroke). A symptomatic postoperative stroke occurred in 42 (16.8%) of our cohort. Eight thereof were non-disabling (3.3%), whereas 34 (13.6%) were disabling strokes. The most frequently affected region was the arteria cerebri media. Embolism was the primary underlying mechanism (*n* = 31; 73.8%). Mortality in patients with postoperative stroke was 26.2%. Logistic regression analysis revealed age over 75 (odds ratio = 3.25; 95% confidence interval 1.20–8.82; *P* = 0.021), a bovine arch (odds ratio = 4.96; 95% confidence interval 1.28–19.28; *P* = 0.021) and an acute preoperative neurological deficit (odds ratio = 19.82; 95% confidence interval 1.09–360.84; *P* = 0.044) as predictors for postoperative stroke.

**CONCLUSIONS:**

Stroke after total aortic arch replacement using the frozen elephant trunk technique remains problematic, and most lesions are of embolic origin. Refined organ protection strategies, and sophisticated monitoring are mandatory to reduce the incidence of postoperative stroke, particularly in older patients presenting an acute preoperative neurological deficit or bovine arch.

**Figure:**
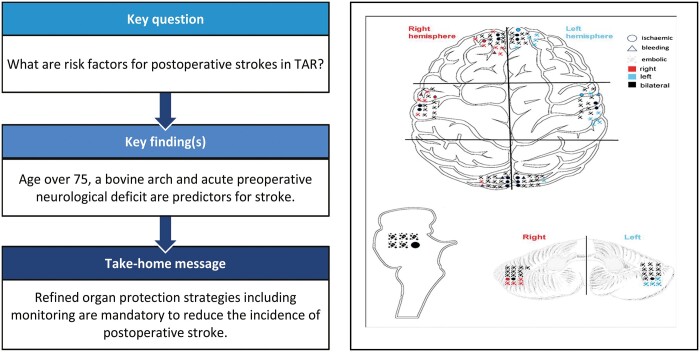

## INTRODUCTION

Total aortic arch replacement (TAR) using the frozen elephant trunk (FET) technique was introduced in 2003 [1], and it has since been refined: for one, off-the-shelf hybrid prostheses became available in different modifications and lengths, and secondly, surgical techniques improved as zone 2 distal anastomosis simplified implantation and lowered the incidence of postoperative spinal cord injuries [2, 3]. Accordingly, short- and long-term survival improved over time, but aortic reinterventions are frequently needed [4]. Despite these refinements, stroke-induced neurological impairments remain a major postoperative complication whose mechanisms have neither been adequately analysed nor understood—nor has the stroke rate fallen over the last decade [3].

Aim of this study was to analyse risk factors for postoperative stroke, evaluate its underlying mechanisms and report on the outcome of patients with postoperative stroke after TAR using the FET technique.

## PATIENTS AND METHODS

### Ethics statement

IRB approval was obtained on 4 February 2021 (No. 20-1302) by the University of Freiburg’s institutional review board and informed consent was waived.

### Patients

Two-hundred and fifty consecutive patients underwent TAR using the FET technique at the University Heart Centre Freiburg between March 2013 and November 2020 for acute and chronic aortic pathologies including acute and chronic aortic dissection, degenerative aneurysm and penetrating aortic ulcer.

### Data collection and definition of parameters

Data were collected retrospectively from our centre’s database. Stroke was classified according to the VARC-2 criteria using the modified Rankin scale (mRS) and subclassified as disabling stroke (mRS > 2) and non-disabling stroke (mRS < 3) [5]. A procedure-related stroke was defined as new postoperative deficit without neurological symptoms within 30 days before surgery. The others were defined as disease-related. An acute preoperative neurological deficit occurred within 30 days before surgery. Postoperative strokes were interdisciplinarily evaluated by a cardiac surgeon, neurologist and radiologist, and subclassified to the respective cerebral territory (frontal, parietal, posterior, cerebellar, stem or intracranial bleeding) and to the assumed stroke mechanism (embolic, perfusion/haemodynamic, microangiopathy). Our multidisciplinary team discussed inconsistencies and then made the decision.

### Preoperative assessment

Despite computed tomography angiography (CTA) of the aorta starting in 2018, elective patients underwent additional colour-coded duplex sonography of the carotid arteries and a concomitant or additional computed tomography angiography scan to identify any variants in the circle of Willis that could influence the intraoperative perfusion strategy. A preoperative, new-onset neurological impairment including coma, or a supra-aortic vessel occlusion caused by the acute aortic pathology was not considered reasons to withhold surgery.

### Surgical technique

Our surgical technique has been described in detail [2, 6–8]. We normally implant the short version of the Thoraflex hybrid-graft (100 mm; Terumo Aortic, Inchinnan, UK); distal anastomoses were routine in zone 2 following zone 3 anastomoses until 2015. The axillary artery is our standard access vessel for arterial cardiopulmonary bypass inflow even if there is a vessel dissection, and all surgeries were performed through a full median sternotomy. Our intended core body temperature is 25 degrees. Bilateral selective antegrade cerebral perfusion (SACP) is our standard cerebral perfusion technique (10 ml/kg/min). Nevertheless, in case of a hypoplastic right vertebral artery, or if the left vertebral artery ends isolated without forming the basilar artery, we usually employ trilateral cerebral perfusion via additional cannulation of the left axillary artery. Bifrontal near-infrared spectroscopy (NIRS) is routine to monitor cerebral oxygenation. We apply cold blood cardioplegia or the beating-heart technique using 300 ml normothermic myocardial perfusion for myocardial protection [7].

### Statistical analysis

All values are expressed as number (percentage) or median (first quartile; third quartile). IBM SPSS Statistics 27 for Macintosh (Armonk, NY, USA) was used for statistical analysis. Normality was assessed graphically using Q–Q plots. Group comparison for the univariable analysis was performed using Student’s *t*-test for continuous variables. For categorical variables, the Chi-squared or Fisher’s Exact test was applied when appropriate. A logistic regression analysis was done to identify independent predictors for postoperative stroke. We selected these clinical covariates and entered them in the model: age over 75, acute type A aortic dissection, dissected supra-aortic vessels, bovine arch, SACP time, cardiopulmonary bypass time, zone 2 distal anastomosis, trilateral cerebral perfusion, preoperative circle of Willis scan, axillary cannulation via Dacron graft, surgery before 2018 and acute preoperative neurological deficit and renal-failure history. The Hosmer–Lemeshow test was used to determine the model’s goodness of fit.

## RESULTS

### Patients characteristics

TAR was performed in 250 patients (64.4% male). Previous strokes were documented in 34 cases (13.6%) at a 39-month interval [IQR 2–92]. Five patients (2%) were referred comatose, and 12 suffered from persisting hemiparesis; both conditions were more frequent in patients with postoperative stroke. Previous aortic interventions or surgeries were frequently observed. Patients characteristics are summarized in Table 1.

**Table 1:** Descriptive characteristics of the cohort

	Total	No stroke	Stroke	*P*-value
Demographics	*n* = 250	*n* = 208	*n* = 42	
Age (years)	67 [59–74]	67 [59–74]	71 [59–76]	0.11
Male	161 (64.4)	133 (63.9)	28 (66.7)	0.86
Chronic health conditions and risk factors				
Hypertension	215 (86.0)	179 (86.1)	36 (85.7)	>0.99
COPD	26 (10.4)	22 (10.6)	4 (9.5)	>0.99
Cardiac tamponade	9 (3.6)	5 (2.4)	4 (9.5)	0.047
Coronary artery disease	74 (29.6)	61 (29.3)	13 (31)	0.85
Chronic renal impairment	33 (13.2)	25 (12.0)	8 (19)	0.21
Bicuspid aortic valve	11 (4.4)	10 (4.8)	1 (2.4)	0.70
Connective tissue disorder	23 (9.2)	22 (10.6)	1 (2.4)	0.14
Preoperative neurological state				
History of stroke	34 (13.6)	22 (10.6)	12 (28.6)	0.004
Time of previous stroke (months)	39 [2–92]	40 [10–81]	25 [0–210]	0.75
Coma	5 (2.0)	3 (1.4)	2 (4.8)	0.13
Hemiparesis	12 (4.8)	7 (3.4)	5 (11.9)	0.018
Previous cardiac or aortic procedures				
Time of previous intervention (years)	5 [1–10]	5 [1–10]	5 [3–11]	0.69
Coronary artery bypass grafting	8 (3.2)	7 (3.4)	1 (2.4)	0.99
Aortic valve replacement	28 (11.2)	24 (11.5)	4 (9.5)	0.80
Mitral valve repair	1 (0.4)	1 (0.5)	2 (4.8)	0.99
Ascending/hemiarch replacement	78 (31.2)	69 (33.2)	9 (21.4)	0.20
Other	52 (20.8)	48 (23.1)	4 (9.5)	0.090
Aortic re-do	95 (38.0)	85 (40.9)	10 (23.8)	0.037
Re-sternotomy	84 (33.7)	68 (32.7)	9 (21.4)	0.15

Data are presented as median (first quartile; third quartile) or as number (%).

COPD: chronic obstructive pulmonary disease.

### Aortic characteristics

Most had undergone surgery for chronic aortic dissections; acute type A aortic dissection was the indication in 35 patients (14%). We identified no differences in underlying pathologies in both groups. Aortic arch variants were observed in 36 (14.4%) patients [isolated offspring of the left vertebral artery *n* = 10 (4%), bovine arch *n* = 14 (5.6%) and an aberrant right subclavian artery *n* = 5 (2%)]. Patients with postoperative stroke were more likely to present a bovine arch (14.3% vs 3.8%; *P* = 0.012). In those with aortic dissection, the dissection membrane extended into the supra-aortic and extracranial vessels in most patients (*n* = 81; 51.9%), and we diagnosed an occlusion in 16 (10.3%) patients. Computed tomography angiography data are summarized in Table 2.

**Table 2:** Computed tomography data

Underlying pathologies	Total	No stroke	Stroke	*P*-value
	*n* = 250	*n* = 208	*n* = 42	
Acute aortic dissection				
Type A	35 (14)	26 (12.5)	9 (21.4)	0.14
Type B	22 (8.8)	17 (8.2)	5 (11.9)	0.39
Non-A non-B	20 (8.0)	17 (8.2)	3 (7.1)	>0.99
Chronic aortic dissection				
Residual type B dissection after type A repair	55 (22)	50 (24)	5 (11.9)	0.10
Type B	14 (5.6)	13 (6.3)	1 (2.4)	0.48
Non-A non-B	10 (4.0)	10 (4.8)	0 (0.0)	0.22
Aneurysm	72 (28.8)	57 (27.4)	15 (35.7)	0.35
PAU	21 (8.4)	18 (8.7)	3 (7.1)	0.78
Others	2 (0.8)	1 (0.5)	1 (2.4)	0.31
Aortic arch variants	36 (14.4)	26 (12.5)	10 (23.8)	0.043
Isolated offspring left vertebral artery	10 (4)	9 (4.3)	1 (2.4)	0.71
Bovine arch	14 (5.6)	8 (3.8)	6 (14.3)	0.012
Aberrant right subclavian artery	5 (2.0)	2 (1.0)	3 (7.1)	0.029
Stenosis of extracranial vessels	28 (11.2)	27 (13)	1 (2.4)	0.050
Dissection’s extension in patients with aortic dissection	*n* = 156	*n* = 133	*n* = 23	
Supra-aortic and extracranial vessels	81 (51.9)	69 (51.9)	12 (52.2)	0.71
Brachiocephalic trunk	49 (31.4)	39 (29.3)	10 (43.5)	0.27
Right subclavian artery	15 (9.6)	12 (9.0)	3 (13.0)	0.71
Right common carotid artery	27 (17.3)	21 (15.8)	6 (26.1)	0.27
Left common carotid artery	38 (24.4)	32 (24.1)	6 (26.1)	>0.99
Left subclavian artery	55 (35.3)	47 (35.3)	8 (34.8)	>0.99
Left vertebral artery	4 (2.6)	4 (3.0)	0 (0.0)	>0.99
Right vertebral artery	1 (0.6)	1 (0.8)	0 (0.0)	>0.99
Thoracic descending aorta	137 (87.8)	117 (88)	20 (87)	0.39
Abdominal aorta	111 (71.2)	95 (71.4)	16 (69.6)	0.49
Iliac arteries	86 (55.1)	72 (54.1)	14 (60.8)	>0.99
Occluded supra-aortic and extracranial vessels	16 (10.3)	10 (7.5)	2 (8.7)	0.70
Right common carotid artery	4 (2.6)	3 (1.4)	1 (2.4)	0.66
Left common carotid artery	4 (2.6)	3 (1.4)	1 (2.4)	0.66
Left subclavian artery	1 (0.6)	1 (0.5)	0 (0.0)	>0.99
Left vertebral artery	2 (1.3)	2 (1.0)	0 (0.0)	>0.99
Right vertebral artery	5 (3.2)	5 (2.4)	0 (0.0)	>0.99

Data are presented as number (%).

PAU: penetrating aortic ulcer.

### Surgical characteristics

Concomitant procedures were performed in 99 patients (39.6%), with aortic valve replacement the most common (*n* = 39; 15.6%). We detected no differences regarding concomitant procedures between patients with and without postoperative stroke. We applied beating-heart technique for myocardial protection in 56 patients (22.4%)—more often in those without stroke. Cardiopulmonary bypass and SACP time were significantly longer in patients with stroke. Indications for aortic valve replacement were moderate or severe aortic stenosis in 2 (0.8%) and 5 (2.0%) patients, respectively, as well as moderate or severe aortic regurgitation in 19 (7.6%) and 13 (5.2%) patients, respectively. Surgical characteristics are summarized in Table 3.

**Table 3:** Surgical characteristics of the cohort

	Total	No stroke	Stroke	*P*-value
	*n* = 250	*n* = 208	*n* = 42	
Concomitant cardiac and vascular procedures	99 (39.6)	81 (38.9)	18 (42.9)	0.73
Aortic root conduit	19 (7.6)	17 (8.2)	2 (4.8)	0.75
Valve-sparing aortic root replacement	16 (6.4)	14 (6.7)	2 (4.8)	>0.99
Aortic valve replacement	39 (15.6)	31 (14.9)	8 (19.0)	0.64
Coronary artery bypass grafting	35 (14.0)	27 (13.0)	8 (19.0)	0.33
Thoracic endovascular aortic repair	9 (3.6)	6 (2.9)	3 (7.1)	0.18
Intraoperative data				
Operation time (min)	384 [328–445]	374 [320–431]	417 [360–541]	0.005
CBP time (min)	209 [174–247]	203 [173–245]	228 [185–276]	0.028
Cross-clamp time (min)	119 [91–152]	115 [90–151]	131 [97–170]	0.094
Bilateral cerebral perfusion	188 (75.2)	155 (75.2)	33 (78.6)	>0.99
Trilateral cerebral perfusion	30 (12.0)	23 (11.2)	7 (16.7)	0.43
SACP time (min)	70 [50–112]	65 [49–104]	93 [70–147]	0.020
Lowest body temperature (degrees celsius)	24.8 [24–25.4]	24.8 [24–25.5]	24.5 [23.6–25]	0.014
Beating-heart technique	56 (22.4)	53 (25.5)	3 (7.1)	0.008

Data are presented as median (first quartile; third quartile) or as number (%).

CPB: cardiopulmonary bypass; min: minutes; SACP: selective antegrade cerebral perfusion.

### Clinical outcome and follow-up

In-hospital mortality was 10%. A symptomatic postoperative stroke occurred in 16.8% of our cohort. Thirty-three (13.2%) were procedure-related, whereas 2.8% (*n* = 7) were disease-related or occurred during hospital stay (*n* = 2; 1 embolic stroke and 1 patient after cardiopulmonary resuscitation). Clinical outcomes and follow-up data are in Table 4. Seven of the procedure-related strokes were non-disabling (2.8%), whereas 26 (10.4%) were disabling strokes. The most frequent stroke-affected area was the arteria cerebri media territory, and embolism was the main underlying mechanism (*n* = 31; 73.8%). Mortality in patients suffering a postoperative stroke was higher (26.2%; *n* = 11), and they also underwent tracheostomy, dialysis and prolonged ventilation more often. Two of our 5 comatose patients suffered a disabling stroke (mRS 4) and the others died in hospital. Incidence of stroke in patients with bovine arch was 42.9% (50% thereof of embolic origin). About 16.7% were left-sided, 33.3% were right-sided and 50% were bilateral strokes. Outcomes of patients with stroke are illustrated in Table 4.

**Table 4:** Clinical outcome and follow-up characteristics of the cohort

Clinical outcome	Total	No stroke	Stroke	*P*-value
	*n* = 250	*n* = 208	*n* = 42	
In-hospital mortality	25 (10.0)	14 (6.7)	11 (26.2)	0.001
Stroke	42 (16.8)			
Procedure-related	33 (13.2)		33 (78.6)	
Disabling	26 (10.4)		26 (61.9)	
Non-disabling	7 (2.8)		7 (16.7)	
Disease-related or during hospital stay	9 (3.6)		9 (21.4)	
Disabling	8 (3.2)		8 (19.1)	
Non-disabling	1 (0.4)		1 (2.4)	
mRS				
0			2 (4.8)	
1			6 (14.3)	
2			2 (4.8)	
3			7 (16.7)	
4			8 (19)	
5			7 (16.7)	
6			10 (23.8)	
Cerebral territory^a^				
Left-sided			8 (19.0)	
Right-sided			13 (30.9)	
Bilateral			21 (50.0)	
Frontal			32 (73.8)	
Parietal			30 (71.4)	
Posterior			20 (47.6)	
Cerebellar			20 (47.6)	
Stem			7 (16.7)	
Intracranial bleeding			6 (14.3)	
Assumed stroke cause				
Embolic			31 (73.8)	
Perfusion/haemodynamic			6 (14.3)	
Microangiopathy			1 (2.4)	
Undetermined			4 (9.5)	
Symptomatic SCI	3 (1.2)	2 (1.0)	1 (2.3)	0.43
Bleeding	37 (14.8)	32 (15.4)	5 (11.9)	0.64
Dialysis	31 (12.4)	20 (9.6)	11 (26.2)	0.008
Ventilation time (hours)	21 [15–48]	21 [15–39]	43 [19–132]	0.012
Tracheostomy	20 (8)	12 (5.8)	8 (19.0)	0.009
ICU stay (days)	6 [4–12]	6 [3–10]	12 [6–19]	<0.001
Hospital stay (days)	17 [13–24]	17 [13–24]	19 [8–24]	0.84
Follow-up data	*n* = 225	*n* = 194	*n* = 31	
Follow-up (years)	1.2 (0.3–2.8)	1.5 (0.4–2.9)	0.7 (0.2–2.4)	
Follow-up mortality	23 (10.2)	20 (9.6)	3 (7.1)	0.78
Aortic reintervention	78 (34.7)	73 (35.1)	5 (16.1)	0.020
Open surgery	4 (1.8)	4 (1.9)	0 (0.0)	0.70
Endovascular extension	63 (28)	59 (28.4)	4 (12.9)	0.003
Hybrid approach	11 (4.9)	10 (4.8)	1 (3.2)	>0.99

Data are presented as median (first quartile; third quartile) or as number (%).

a Multiple nomination possible.

ICU: intensive care unit; mRS: modified Rankin scale; SCI: spinal cord injury.

### Logistic regression analysis

As predictors for postoperative stroke, our logistic regression model identified: age over 75 (odds ratio = 3.25; 95% confidence interval 1.20–8.82; *P* = 0.021), a bovine arch (odds ratio = 4.96; 95% confidence interval 1.28–19.28; *P* = 0.021) and acute preoperative neurological deficit (odds ratio = 19.82; 95% confidence interval 1.09–360.84; *P* = 0.044) as predictors for postoperative stroke. The full model is shown in Table 5.

**Table 5:** Logistic regression model for stroke

Logistic regression model	Odds ratio	95% CI	*P*-value
Acute preoperative neurological deficit	19.82	(1.09–360.84)	0.044
Age over 75 years	3.25	(1.20–8.82)	0.021
Trilateral cerebral perfusion	3.43	(0.97–12.18)	0.057
Zone 2 distal anastomosis	0.82	(0.29–2.35)	0.71
Bovine arch	15.19	(1.30–177.84)	0.021
Supra-aortic vessel dissection	0.69	(0.23–2.12)	0.26
Axillary cannulation via dacron graft	1.44	(0.54–3.84)	0.47
Surgery before 2018	1.95	(0.59–6.47)	0.27
Acute type A aortic dissection	3.18	(0.90–11.22)	0.073
Cardiopulmonary bypass time	1.01	(0.99–1.01)	0.24
Selective antegrade cerebral perfusion time	0.99	(0.98–1.01)	0.41
History of renal failure	1.57	(0.45–5.45)	0.48
Preoperative Circle of Willis scan	1.15	(0.28–4.68)	0.85

CI: confidence interval.

## DISCUSSION

Stroke after TAR using the FET technique remains problematic, and most lesions are of embolic origin. Refined organ protection strategies and sophisticated monitoring are mandatory to reduce the incidence of postoperative stroke, particularly in older patients with an acute preoperative neurological deficit or bovine arch.

Although our cohort’s demographics and medical histories are in line with other large FET trials, our patients seem older than in other studies [9, 10]. Our study reveals that an acute preoperative neurological deficit is a predictor for a perioperative one. Other trials, and a large nationwide analysis after cardiac or aortic surgery in the USA also arrived at this finding [11]. These are patients tending to have widespread cerebrovascular disease, whose cerebral blood flow may be impaired and likely to trigger atherosclerotic or thrombotic embolism [12]. This fact is also supported by our results, as the stroke incidence was notably higher in patients with degenerative aortic aneurysms than with chronic aortic dissections. However, another working group that investigated the postoperative stroke risk dependent on the presence or not of previous embolism in patients with infective endocarditis detected no differences in neurological outcome [13]. Since these strokes occurred in different territories, the authors attributed the procedure and manipulation themselves as having played the primary role. Note that we were unable to enter coma in our analysis due to the small number of patients. Nevertheless, another study showed that patients suffering from coma due to acute type A aortic dissection revealed acceptable clinical outcomes provided they had undergone surgery immediately (mortality was 14%, 86% of the cohort recovered consciousness and the majority could engage in daily activities independently [14]).

Acute type A aortic dissection was not predictive for postoperative stroke in our study. Nevertheless, our selection of patients with acute type A aortic dissection contributes to the numerically higher stroke incidence in these patients since only those with an arch-entry tear or supra-aortic vessel occlusion undergo TAR in our centre, whereas the others undergo hemiarch replacement. Controversially, our logistic regression model revealed that dissection of the supra-aortic or extracranial vessels was not significant—potentially attributable to the few patients with acute type A aortic dissections. In contrast to our findings, there is evidence that a common carotid artery dissection and carotid true lumen flow impairment are predictive for stroke, but not mortality [15, 16]. Our experience is that dissected supra-aortic vessels do not influence outcomes or cause cannulation difficulties in the right axillary artery when done routinely. Interestingly, a bovine arch has proven to be predictive for postoperative stroke. Dumfarth *et al.* and Kreibich *et al.* made this finding, but no conclusive explanation for bovine arch being predictive for postoperative strokes [17, 18] . Right axillary cannulation might contribute to a higher incidence of embolic strokes especially in patients with acute type A aortic dissection. Consequently, the San Donato group founded the CILCA registry to further investigate the bovine arch’s relevance for postoperative strokes. Another frequent, extremely important aortic arch variant is the isolated offspring from a vertebral artery with a notably variable course. Our clinical observations have revealed a unilateral hypoplastic vertebral artery as often the sole supplier of blood to ‘its’ isolated cerebral region—often without visible collaterals, which is why we have all our elective patients undergo preoperative CT diagnostics of the Circle of Willis. This procedure is facilitated by data from a trial showing that 50% of their patients presented aortic arch anomalies, and in 9 thereof, hypoperfusion could have been caused by unilaterally SACP. This affects the left posterior communication artery especially, as it revealed aplasia or hypoplasia in 35.6% of these patients [19]. Trilateral cerebral perfusion via an additional cannula for the left axillary artery may be necessary in these patients even though it was not predictive in our regression analysis.

Intraoperative cerebral perfusion and hypothermia are key to sustaining cerebral protection during aortic arch replacement. While the antegrade manner of perfusion is widely used, there is ongoing debate as to whether uni-, bi- or trilateral selective cerebral perfusion is ideal. Of note, Zierer *et al.* [20] demonstrated that bilateral SACP correlated with a higher stroke incidence in elective cases (acute type A aortic dissections were excluded) with regard to more manipulation, other researchers observed no significant difference in neurological outcomes between uni- and bilateral perfusion techniques in patients with acute type A aortic dissection [21]. They detected perfusion safety functionally via transcranial Doppler [22]. Moreover, the efficacy of monitoring intracerebral oxygenation by relying on adequate blood perfusion remains debatable. Most centres in Europe use NIRS. But studies have shown that NIRS does not sufficiently monitor the entire cortex’s oxygenation—rather, only a small part of the frontal lobe. NIRS may therefore only indicate a cannula’s malposition or regional hypoperfusion—it cannot detect embolic events. There is no solid evidence that it predicts any postoperative neurological events [23]. In fact, it seems that we currently lack the means to monitor cerebral perfusion adequately despite the obvious urgency of doing so. Our group is currently evaluating the clinical benefits of multichannel NIRS. With this tool, more than 50 electrodes demonstrate the oxygenation of the entire cortex including the posterior territory.

The largest meta-analysis to date of studies (covering 3154 patients) reporting on post-FET procedure outcomes revealed a 7.6% incidence of postoperative stroke, but failed to identify any potential risk factors for stroke after total arch replacement. A major limitation in their study is the study design with limited access to essential variables. Other single-centre studies or registries reported an incidence of 7–11.6% [9, 10, 24, 25]. Of note, they often only mention permanent deficits, and their stroke definitions are not comparable to our study’s. If one made a comparison our procedure-related disabling stroke incidence 10.4% would be the most applicable. Nevertheless, we perceive a slight difference in postoperative stroke rates that may have something to do with the prostheses’ design (island vs multi-branch prosthesis) leading to more surgical manipulation (supported by the numerous embolic strokes in our study). However, other influencing factors, especially technical or perfusion differences among centres, cannot be ruled out [9, 10, 24].

### Limitations

This is a retrospective single-centre study with all the limitations associated with such a study design. Therefore, we could only analyse our protection strategy, and were unable to compare different hypothermic or perfusion settings. As no NIRS data were collected, no analysis of a drop in saturation was possible. No primary analysis was defined. Many tests were conducted without adjusting for multiple testing. Therefore, analyses were exploratory in nature and inferences drawn from them may not be reproducible.

## CONCLUSIONS

Stroke after TAR using the FET technique remains a difficult issue. Whereas most lesions in our patients were of embolic origin, we identified no clear distribution pattern corresponding to their cerebral territory. An acute preoperative neurological deficit, a bovine arch and age over 75 years were our cohort’s only risk factors for postoperative stroke. No morphology or procedure-related factors contributed to the postoperative stroke risk. Refined organ protection strategies and sophisticated monitoring are mandatory in order to reduce stroke after TAR using the FET technique.

**Conflict of interest**: Martin Czerny and Bartosz Rylski are consultants to Terumo Aortic and shareholders of Ascense Medical, Martin Czerny is consultant to Medtronic, Endospan and NEOS, received speaking honoraria from Cryolife-Jotec and Bentley and is shareholder of TEVAR Ltd. 

**Data availability statement:** The data underlying this article cannot be shared publicly due to our institutional review board’s requirements. The data will be shared on reasonable request to the corresponding author.

## Notes

Presented at the 35th Annual Meeting of the European Association for Cardio-Thoracic Surgery, Barcelona, Spain, 13–16 October 2021.

## ABBREVIATIONS

FET: Frozen elephant trunk
mRS: Modified Rankin scale
NIRS: Near-infrared spectroscopy
SACP: Selective antegrade cerebral perfusion
TAR: Total aortic arch replacement
